# MRCK: a master regulator of tissue remodeling or another ‘ROCK’ in the epithelial block?

**DOI:** 10.1080/21688370.2021.1916380

**Published:** 2021-05-19

**Authors:** Ceniz Zihni

**Affiliations:** UCL Institute of Ophthalmology, Department of Cell Biology, University College London, London, UK

**Keywords:** Rho GTPase, MRCK, ROCK, polarity, morphogenesis, cancer

## Abstract

The epithelium forms a smart barrier to the external environment that can remodel whilst maintaining tissue integrity, a feature important for development, homeostasis, and function. Its dysregulation can lead to diseases ranging from cancer to vision loss. Epithelial remodeling requires reorganization of a thin sheet of actomyosin cortex under the plasma membrane of polarized cells that form basolateral contacts with neighboring cells and the extracellular matrix (ECM). Rho GTPases act as spatiotemporal molecular switches in this process, controlling localized actomyosin dynamics. However, the molecular mechanisms that control actomyosin dynamics at the apical cortex are poorly understood. This review focusses on a growing body of evidence that suggest myotonic dystrophy kinase-related Cdc42-binding kinase (MRCK) plays a conserved role in morphogenetic signaling at the apical cortex in diverse cell and tissue remodeling processes. The possible molecular and mechanistic basis for the diverse functions of MRCK at the apical pole will also be discussed.

## Introduction

**Figure 1.**
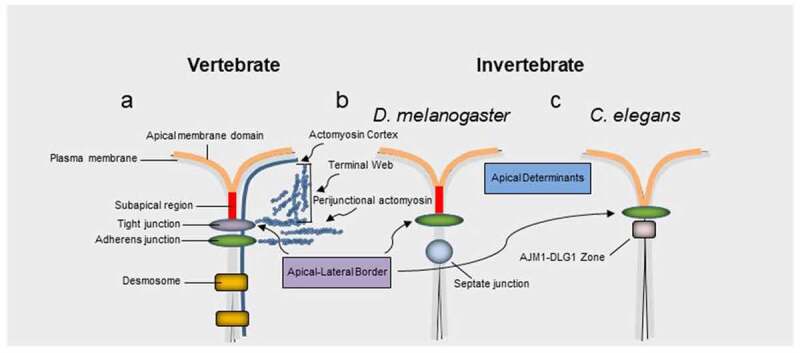
Apical junctions across vertebrates and invertebrates. a, In vertebrates tight junctions are positioned apical to adherens junctions. b, c, Invertebrate adherens junctions are often the most apically positioned junctional structure. The equivalent structure to tight junctions in many invertebrate epithelia, such as *D. melanogaster*, the septate junction, is positioned basal to the adherens junction. Aside from regulating barrier properties apical junctions also play an important role in apicobasal polarity via at least two protein complexes formed by the partitioning defective 3 (PAR3)-PAR6-atypical protein kinase (aPKC)-Cdc42 complex and the protein crumbs homologue 3 (CRB3)-protein associated with Lin-7 1 (PALS1)-PALS1-associated tight junction (PATJ) complex. In vertebrates these complexes have traditionally been associated with tight junctions and their homologues in *D. melanogaster* associate with a subapical region (SAR) apical to adherens junctions. However, an equivalent signaling zone has been proposed in vertebrates.^4,5^ Note, the actin cortex, filamentous actin of the perijunctional belt and terminal web are highlighted in blue. The apical junction molecule 1 (AJM1)–discs large homologue 1 (DLG1) complex^6^ in *C. elegans* is involved in barrier function, similar to tight junctions in vertebrates and septate junctions in many invertebrates

Epithelial cells polarize by developing distinct cell surface domains with different biochemical compositions and functions^1^. The apical membrane domain in vertebrate epithelia is defined by the position of the upmost apical portion of tight junctions. These multiprotein complexes have been implicated in a variety of processes in health and disease, beyond their tradition barrier function.^2,3^ Tight junctions form an apical junctional complex with adherens junctions (Figure 1a) and are associated with the apicobasal polarity machinery.^7^ Invertebrates such as *D. melanogaster* and *C. elegans* contain adherens junctions as the most apical junctional structure^2^ (Figure 1b,c). The apical junctional complex, contributes to the cytoskeleton via an F-actin perijunctional belt and, in highly apically differentiated epithelia, an F-actin rich structural network known as the terminal web (Figure 1a).^2,3,8^ The terminal web is normally linked to specialized organ specific brush border membranes. Evidence also suggests that similar to invertebrate *D. melanogaster*, vertebrate epithelia also contain a subapical domain above the highest positioned junctional structure^4,5^ (Figure 1a,b).

Rho GTPases are critical components, of signaling pathways that regulate the cytoskeleton, to guide diverse cellular functions including proliferation, migration, adhesion, polarization, and specialization of the apical plasma membrane.^3^ This is due to their role as localized molecular switches that cycle between active GTP bound and inactive GDP bound states to spatiotemporally control cell and tissue morphology. The active GTP-bound state allows association with an effector protein that regulates cytoskeletal reorganization to drive cell and tissue morphogenesis.^9,10^ Effector proteins include Neural Wiskott-Aldrich syndrome protein (N-WASP), the p21-activated kinase (PAK), Rho-associated kinase (ROCK) and closely related MRCK. The diverse functions of Rho GTPases, despite their relatively low number is due to the vast number of their regulators that include guanine nucleotide exchange factors (GEFs) that promote the exchange of GDP for GTP, and GTPase-activating proteins (GAPs) that stimulate GTP hydrolysis. These regulators possess distinct localization profiles that largely define the molecular switch properties of Rho GTPases.

The functions of various Rho GTPase signaling pathways at cell-cell and cell-extracellular matrix (ECM) adhesions have been extensively studied.^2,11–13^ However, the role(s) and significance of Rho GTPase signaling at the apical membrane domain, during cell and tissue morphogenesis, is poorly understood. Over the past few years, a growing body of evidence support a conserved role for the Cdc42 effector MRCK as an apical driver of morphogenesis in different developmental and homeostatic contexts.

### Structure of MRCK

**Figure 2.**
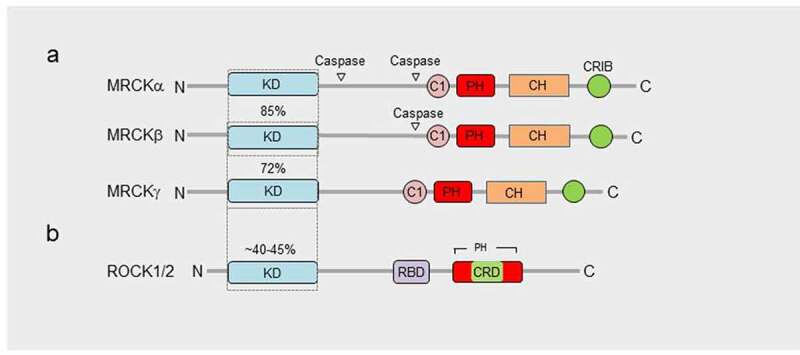
Domain organization of MRCKs and ROCK1/2. a, Homology within the MRCKs is observed over N-terminal kinase domains (KD), C-terminal C1, citron homology domain (CH), pleckstrin homology domain (PH) and Cdc42- and Rac-interactive binding domain (CRIB). b, The N-terminal kinase domain of ROCK1/2 displays significant homology with the MRCKs and has several common substrates (refer to Figure 5). The C-terminal domain of ROCK1/2, that is far less homologous to the MRCKs, includes a Rho-binding domain (RBD), cysteine-rich domain (CRD), different spatially organized PH domain, and lacks C1, CH and CRIB domains

**Figure 3.**
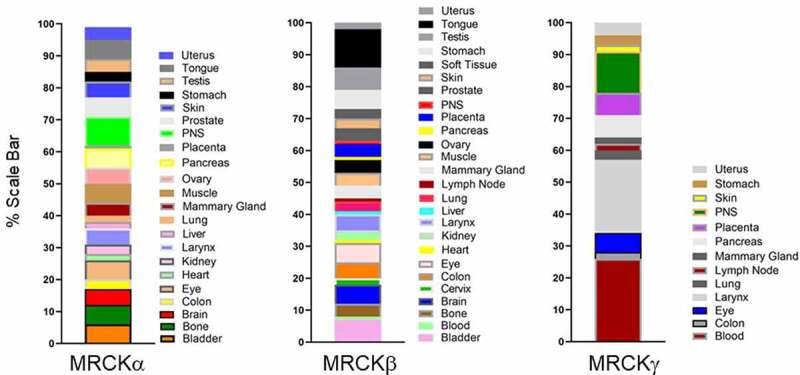
Tissue distribution of MRCKs. MRCKα and β are ubiquitously expressed, whereas MRCKγ is restricted to fewer tissues. The blood, larynx, and peripheral nervous system are indicated to have the highest levels of MRCKγ expression. Determination of relative tissue distributions of MRCKs has been previously described.^16^

There are 3 related MRCK proteins MRCKα, β and γ that are part of a PKA, G, C (AGC) superfamily^14^ (Figure 2a). MRCKα and β, both ubiquitously expressed^15^ (Figure 3), display the most homology with 85% amino acid similarity across their kinase domains and 61% total amino acid identity (Figure 2a).^16^ MRCKγ, which may be restricted to fewer tissues^16,17^ (Figure 3), is closest to MRCKβ with 72% amino acid identity over its kinase domain and 44% identity over the total amino acid sequence (Figure 2a).^18^ Although MRCK proteins were largely identified on the basis of their binding properties to Cdc42-GTP, subsequent studies have demonstrated that MRCKs can act as a Cdc42 and/or Rac effector via their Cdc42/Rac interactive binding (CRIB) domain.^19–21^ The Rho effector ROCK1 and ROCK2 kinases are related to the MRCKs (Figure 2a,b), especially the three-dimensional spatial organization of their kinase domains.^22,23^ Whilst MRCKs carry out their effector functions via their CRIB domains, ROCK kinases possess a Rho-binding domain (RBD) that act downstream of Rho GTPase. In MRCKs the protein kinase C conserved region 1 (C1), at least in the case of MRCKα and MRCKβ, can bind phorbol esters at high affinities^24,25^ that may promote kinase activation^24^ and/or membrane translocation.^25^ The pleckstrin homology (PH)-like domains of MRCKs contain a similar three-dimensional structure and are thought to be important for subcellular localization via binding to other proteins or lipids. MRCKα,β,γ all contain a citron homology (CH) domain that lies adjacent to the PH-like domain.^26^ The spatial arrangement of CH-PH domains is conserved and suggest a cooperative action in possible functions including protein-protein interactions that may contribute to subcellular distribution. It has also been proposed using in vitro assays that the function of Cdc42-GTP may not be to increase kinase activity toward myosin light chain (MLC) but membrane recruitment, bringing MRCK in close proximity to cortical MLC to activate it.^19^

### MRCK as a driver of apical domain organization

#### A. Apical expansion and brush border induction

Polarization requires the segregation of polarity determinants, including partitioning defective (PAR) proteins, into distinct domains.^29,30^ In response to Cdc42 activation the Par6-aPKC complex transiently binds to Par3, in epithelial cells, dissociating from it to demarcate the apical/lateral border, and  segregates into the developing apical domain.^4,31,32^ The apical domain of epithelia often undergoes a morphogenic transformation leading to functional actin-rich structures such as the brush border membrane of absorptive epithelia.

**Figure 4.**
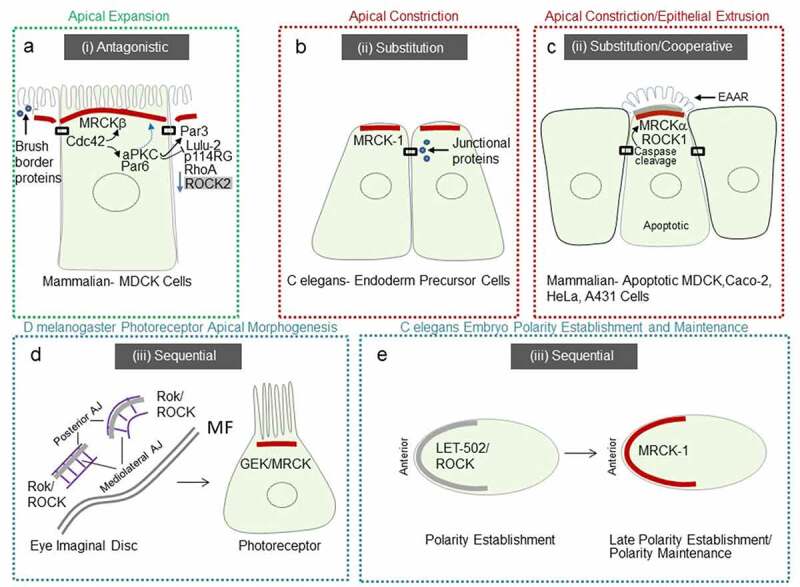
Schematic representation of conserved MRCK activity, at the apical pole

In mammalian MDCK kidney epithelial cells, full specialization of the apical membrane occurs via apical polarity determinants driven by Cdc42 and its apical GEF Dbl3^4^. A hypothetical model of antagonism between Cdc42-driven apical polarity determinants, activated by Dbl3, and RhoA-ROCK2-driven perijunctional actomyosin contractility as a defining step in apical domain organization was previously described.^3^ Recent work substantiates this hypothesis and suggests a role for MRCKβ as a determinant of apical domain organization. Following activation of Cdc42 by Dbl3, recruitment of MRCKβ to the apical cortex promotes polarized activation of myosinII.^27^ MRCKβ recruitment is coupled and coordinated with Par6-aPKCζ apical recruitment to drive a dual effector mechanism (Figure 4a). aPKC is recruited to TJ to deactivate the RhoA/ROCK2 activator p114RhoGEF via inhibition of LULU-2, and phosphorylate Par3 to apically exclude it and define the apical-basolateral border.^4,27,31–33^ Phosphorylation of Par3 by aPKC destabilizes the interaction and results in distribution of Par6-aPKC along an expanding apical membrane domain^4^ a process that is dependent on MRCKβ-activated myosinII.^27^ Apical domain development is concomitant with an apical enrichment of cytosolic components that promote brush border induction.^27,34^

**a**, Mammalian MDCK epithelial cells require MRCKβ at the apical cortex to activate a morphogenetic signaling pathway that works in coordination with another Cdc42 effector Par6, in complex with aPKC. Briefly, apical domain development and cell morphogenesis during polarization require aPKC-dependent deactivation of junctional actomyosin contractility driven by LULU-2-activated p114RhoGEF(p114RG)/RhoA/ROCK2. aPKC also phosphorylates Par3 to apically exclude it, whilst MRCKβ driven apical contractions facilitate separation of Par6-aPKC from junctions into the expanding apical membrane domain. Concomitantly, cytosolic concentration of brush border factors at the apical domain promote microvilli induction. Thus, apical domain expansion and brush border induction result from an *antagonistic* relationship between MRCKβ and ROCK2 signaling at the apical domain (i). **b**, In *C. elegans* internalizing endodermal precursor cells undergo apical constriction. MRCK-1 is required for this process, by playing a role generally associated with ROCK, thus *substitutes* the role of ROCK (ii) by promoting contractility at the apical pole, and enrichment of junctional proteins, to drive apical constriction. **c**, In mammalian epithelial cells MRCKα may *substitute* and/or *cooperate* with ROCK1 (ii), depending on the cell model to promote epithelial cell extrusion via a similar base mechanism to apical constriction. **d**, Developing *D. melanogaster* retina require GEK/MRCK during apical domain development of photoreceptors. Briefly, in the early eye imaginal disc Rok/ROCK but not GEK/MRCK is required for junctional remodeling at line and arc structures. During photoreceptor domain development GEK/MRCK, but not Rok is required for apical morphogenesis. **e**, In the *C. elegans* embryo LET-502/ROCK is required at the anterior pole for polarity establishment, whereas CDC-42-activated MRCK-1 is required for polarity maintenance, and possibly late polarity establishment. Thus, retinal development in *D. melanogaster* and the establishment of polarity by the *C. elegans* embryo require *sequential* activation of ROCK and MRCK (iii). Note, green, red and blue dashed outlines highlight a proposed *antagonistic* (i), *substitution/cooperative* (ii) and *sequential* (iii) spatiotemporal relationship between MRCK and ROCK. Red bars represent MRCK activity and gray bars represent ROCK activity.

#### B. Apical constriction

Apical constriction, an opposing process to apical expansion, describes a shrinking of the apical cell surface and is an important morphogenic event during developmental processes such as neural tube formation in vertebrates and gastrulation in many systems.^35^ Apical constriction involves medio-apical actomyosin networks, positioned at the apical cortex, under tension that generate force^36–38^ and perijunctional actomyosin belts that contract via a purse-string mechanism.^39,40^ Since medio-apical networks are connected to junctions a fundamental question has persisted, as to how these two structures are maintained, coupled, and coordinated spatially and temporally.

The gastrulation movements in the early *C. elegans* embryo have provided a useful model to address this question. Contraction of apical actomyosin networks is required for the internalization of endoderm precursor cells (EPCs).^41,42^ For actomyosin-based contractile force to drive tissue morphogenesis, the force must be mechanically transmitted to adjacent cells. The force-bearing bridge between the actomyosin cortices of neighboring cells is the cadherin-catenin complex (CCC).^43,44^ MRCK-1 is required for the activation of myosin at the apical cortex of gastrulating cells and apical constriction.^45^ MRCK-1 localizes at the apical pole of apically constricting endoderm precursor cells, activating actomyosin contractility, that enriches junctional proteins α-catenin, β-catenin, and cadherin^45^ (Figure 4b). This study indicates that MRCK-1 links developmental patterning mechanisms to cytoskeletal force generation to drive apical constriction.

#### C. Epithelial cell extrusion

Epithelial cells need to maintain a closed sheet during homeostasis. Such a state requires synchronization of the junctional actomyosin belt between neighboring cells that control precise removal of aged or damaged cells by a process called epithelial cell extrusion. Loss of epithelial homeostasis is associated with different diseases, for example, cancer.^3,46^

Actomyosin reorganization occurs in both the apoptotic cell and neighboring cells that coordinate with each other to facilitate removal of the apoptotic cell whilst quickly replacing a potential space.^47–49^ Apoptotic cells use the sphingosine-1-phosphate (S1P) receptor 2 pathway to mediate their status to neighboring cells, resulting in the assembly of a basal actomyosin constriction ring.^50^ However, the physical role of apoptotic cells during epithelial extrusion was less clear until recently.

Apoptosis is triggered by several pathways that converge into a cascade of caspases that drive hallmark features including activation of cytoplasmic endonucleases, release of immunomodulation proteins, nuclear fragmentation, and the formation of blebs and apoptotic bodies.^51–54^ Although caspases were initially proposed to degrade the actin cytoskeleton^55,56^ consequent work indicated that the actin cytoskeleton is the main driving force in apoptotic cells,^57^ being responsible for most of the associated morphological processes.^47,58–61^ Evidence suggests that ROCK1 kinase activity on myosin light chain 2 (MLC2), is increased by caspase 3 driven proteolytic cleavage causing contraction of the cortical actomyosin network that drives blebbing.^59,62^ A subsequent study demonstrated that epithelial extrusion is a biphasic process, involving basal ring constriction that is generally preceded by the formation of a dense apical actin structure.^48^ More recently MRCKα has been identified as a key downstream kinase of cell morphogenesis in the apoptotic pathway, that initiates epithelial extrusion^63^ (Figure 4c). During this process MRCKα is constitutively activated by proteolytic cleavage at aspartate 478 residue. MRCKα activity increases MLC2 phosphorylation at the apical pole which drives the formation of an extrusion apical actin ring (EAAR) in an apoptotic cell. The EAAR structure pulls actin bundles, resulting in cell body compaction and removal, by producing cell-autonomous forces as an early event of epithelial extrusion. In addition to MRCKα, caspase-mediated cleavage irreversibly activates ROCK1^59,62^ and MLCK,^64^ resulting in constitutive phosphorylation of MLC2. Such a ubiquitous activation of myosin is thought to underly a requirement for a rapid and substantial burst of myosin activity.^63^

### MRCKs role in cancer

**Figure 5.**
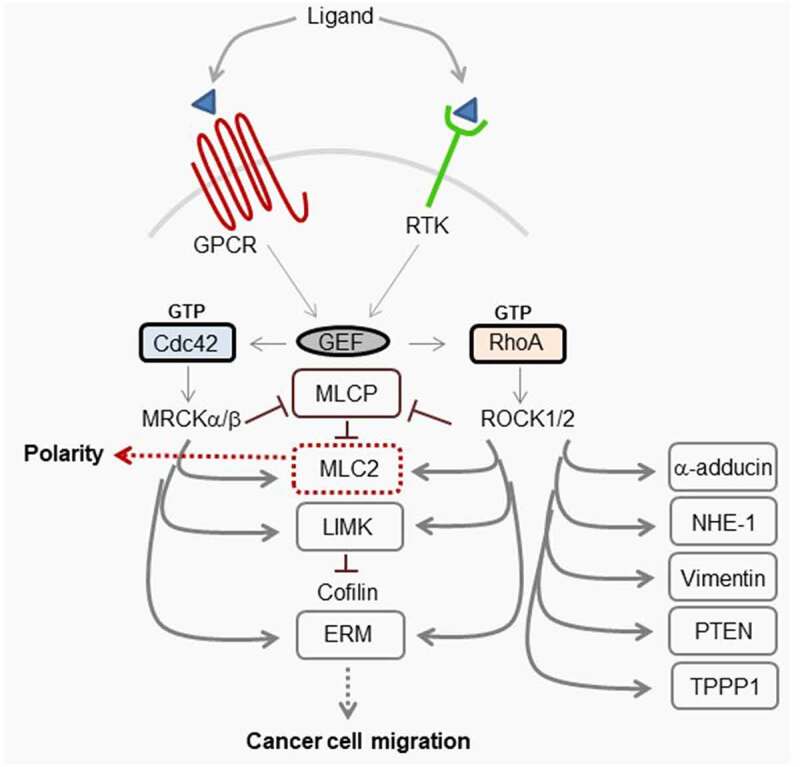
Schematic representation of MRCK and ROCK1/2 signaling

The importance of Cdc42-MRCK and RhoA-ROCK1/2 in both polarity and cell motility (Figure 5) place them as important factors in both normal cellular processes and cancer. MRCK may possess tumorigenic properties due to increased kinase activity,^65^ that may be independent of Cdc42^19^, and/or its overexpression in certain types of tumor.^28^ Additionally, the dysregulation of Cdc42 due to overexpression,^66,67^ or by GEFs, may contribute to the tumorigenic properties of MRCK. Since Cdc42 GEFs are activated by a diverse spectrum of cell surface receptors including G-protein coupled receptors, growth factor receptors, integrins and cytokines it is not surprising that several GEFS have been identified to be dysregulated in cancer.^68^ The ability of Cdc42-signaling to contribute to cell migration would mean that under oncogenic conditions, due to other signaling factors, it could potentially behave as a tumorigenic factor in the absence of its own direct dysregulation. RhoA and its GEFs Vav and Trio are also overexpressed in particular cancers although, as with Cdc42, its inherent ability to function in both migration and polarity means it may act as either a tumorigenic factor or tumor suppressor.^69,70^ Rho-ROCK and Cdc42-MRCK-signaling have been demonstrated to converge during actomyosin-dependent cell motility.^71^ Rho-ROCK is important for tumor cell migration through a three-dimensional matrix with a rounded morphology, whereas Cdc42-MRCK co-operates with Rho-ROCK to generate and maintain elongated morphology and invasion. It is thought that proteins that function in tumor cell invasion and metastasis also contribute to the growth of primary tumors.^72^ Therefore, the development of specific MRCK inhibitors is expected to have beneficial effects on reducing tumor growth and progression^65^

Ligand binding to receptors, such as receptor tyrosine kinases (RTKs) and G-protein coupled receptors, activate guanine nucleotide exchange factors (GEFs) that in turn activate Rho GTPases Cdc42 and RhoA. Their effectors MRCK and ROCK1/2, respectively, can target common substrates that include myosin light chain 2 (MLC2), myosin light chain phosphatase (MLCP), LIM domain kinase (LIMK), and ezrin-radixin-moesin (ERM) family of proteins that can regulate migration of cancer cells. Note, activation of myosinII by MRCK and ROCK2 can also drive epithelial polarization.^3,27^ ROCK also targets distinct substrates including α-adducin, Na+/H+-Exchanger 1 (NHE-1), vimentin, phosphatase and tensin homolog (PTEN), and tubulin polymerization promoting protein 1 (TPPP1).^28^

## Conclusions and future perspectives

The apical membrane of polarized epithelial cells undergo tissue and developmental process-specific modifications that rely on actomyosin based forces to drive shape changes. In recent years studies from a variety of laboratories have identified a conserved role for MRCK in yet diverse apical domain organization processes. The precise role MRCK plays at the apical domain may be partly due to structural characteristics of the protein variants.

It has been proposed that the carboxyl-terminus of MRCK comprised of C1, PH, CH, and CRIB domains may specify its membrane localization in response to extracellular signals. For instance, vasopressin treatment of kidney cells from the collecting duct promotes increased apical plasma membrane localization of MRCKβ.^73^ The bringing of MRCK in close proximity to MLC2, by Cdc42-GTP binding to its CRIB domain,^19^ may therefore bridge localization with functional activity. Since the spatial organization of the carboxyl-terminus CH-PH domains is conserved and flanked by a CRIB domain in all 3 MRCKs, this may suggest that under certain stimuli MRCKs may possess an inherent capacity to function as a regulator of the apical cortex.^16,27,45,63,73^ Subtle differences between MRCKs may determine functional specificity. For instance, during apoptosis both MRCKα and MRCKβ are cleaved by caspases at their coil-coiled regions.^63^ However, MRCKα contains additional cleavage sequences that may increase its susceptibility to caspase cleavage (Figure 2a), defining its distinct role in apoptosis (Figure 4c). Intriguingly, apical localization of MRCKα in mammalian epithelial cells was functionally enhanced toward MLC2 by caspase cleavage and removal of its carboxyl-terminus. Thus, additional regions of MRCK may contribute to its localization.

Another factor that is likely to define the precise role of MRCK at the apical pole is its spatiotemporal relationship to ROCK1/2. For instance, both MRCKα and *C. elegans* MRCK-1 regulate apical constriction^45,63^ (Figure 4b,c). MRCKα is important for apical constriction during apoptosis,^63^ whereas MRCK-1 regulates apical constriction during gastrulation.^45^ In *C. elegans* MRCK-1 seems to function by substituting the role of ROCK, to drive actomyosin contractility at the apical pole and the enrichment of junctional proteins that may modulate force transmission between neighboring cells (Figure 4b). During *D. melanogaster* gastrulation polarized myosin activation is due to Rok/ROCK.^74,75^ In mammalian epithelial cells p114RhoGEF was previously reported to drive junction assembly, and apical constriction, although ROCK was reported to function directly at junctions in these cells.^33,76^ Apical constriction in vertebrates during invagination processes is also dependent on ROCK.^39^ During apoptosis of mammalian epithelial cells, the precise relationship between MRCKα and ROCK1, i.e., cooperative and/or one of substitution, needs further investigation and may depend on the cellular model.^63^ The EAAR structure reported to be mediated by MRCKα activation of myosinII, during apoptosis (Figure 4c), seems to be a distinct structure from apical constriction structures previously described.^63^ The shared localization of the EAAR with MRCK and ROCK may suggest that its mechanism is derived from the base mechanism of apical constriction.

In vertebrate mammalian epithelial cells MRCKβ plays an important role in apical morphogenesis during apical expansion and brush border induction through Cdc42-dependent activation of myosinII.^4,27^ The apical distribution of MRCKβ is conserved with MRCK-1 in *C. elegans* and MRCKα in mammalian epithelial cells and would be expected to drive a similar contractile force-generating process that controls apical constriction. The key difference in the fate of the apical domain seems to be the spatiotemporal relationship between MRCK and ROCK. The cooperative or substitution role MRCKα and MRCK-1 play with ROCK in apoptosis and gastrulation, is contrasted during apical expansion and brush border induction with an antagonistic one. Such an antagonistic behavior may be facilitated by differences between the carboxyl-terminus regions of MRCK and ROCK^77^ (Figure 2a,b), which may be important for orchestrating spatially distinct actomyosin regulatory mechanisms via controlling distinct subcellular recruitment. Indeed, as MDCK epithelial cells undergo polarization and apical specification, MRCKβ is coupled to another Cdc42 effector, complexed Par6-aPKCζ, that downregulates the pro-apical constriction LULU-2-p114RhoGEF-RhoA-ROCK2 pathway localized at the apical junctional complex^27,33,78^ (Figure 4a). Thus, MRCKβ plays a more complex role in apical expansion, both facilitating and defining an expanding apical membrane domain through a similar contractile base mechanism at the apical pole displayed by MRCKα and MRCK-1, yet antagonizing perijunctional contractility. It is notable that both MRCKβ and MRCK-1 promote concentration of cytosolic brush border and junctional proteins, respectively, during their function at the apical domain^27,45^ (Figure 4a,b). Although polarization of subcellular domains may facilitate cytosolic segregation of proteins,^79^ future work is required to precisely understand how apical contractility generated by MRCK drives process specific localization of these factors.

In *D. melanogaster* Genghis Khan (GEK), the orthologue of MRCK, was demonstrated to localize at the apical pole of photoreceptor cells and control actomyosin contractility dependent apical morphogenesis.^27^ In early eye discs Rho-1-Rok/ROCK signaling drives junctional remodeling^80–82^ before Cdc42-GEK activation leads to a reorientation of the actomyosin contractility gradient to control apical morphogenesis^27^ (Figure 4d). Future work to understand the precise mode by which Rok/ROCK activity is superseded by GEK/MRCK activity may provide further insight into the underlying mechanisms of apical domain organization during photoreceptor development in *D. melanogaster*. A similar conservation of a sequential function between RhoA and Cdc42 signaling is observed during polarization of the *C. elegans* embryo. RhoA orthologue RHO-1 is required for the establishment of polarity, whereas CDC-42 is required for polarity maintenance (Figure 4e). Interestingly, CGEF-1, the orthologue of mammalian Dbl3, is the robust activator of CDC-42 that together with a GAP at the posterior pole, CHIN-1, restricts CDC-42 activity to the anterior pole.^83^ Furthermore, polarized distribution of non-muscle myosinII at the anterior pole during the maintenance phase, is achieved by restricting CDC-42 activity and thereby the activity of its effector MRCK-1 at this position. A more recent study indicates that CDC-42 activity at the anterior pole increases during the late establishment phase of polarity, suggesting that MRCK-1 function may be activated during this phase.^84^ Recent work in *D. melanogaster* hair follicle epithelia has implicated GEK/MRCK as a Cdc42 effector at the apical pole. RhoGAP19D, was found to suppress Cdc42 activity at the lateral domain and exclude it to the apical pole.^85^ The orthologue of RhoGAP19D in *C elegans*, PAC-1, carries out a similar function in the early embryo^86^ and was found to be required for polarized activity of MRCK-1 in endodermal precursor cells.^45^ In hair follicle epithelia GEK/MRCK localized laterally in *rho 19d* inactive mutant clones resulting in increased lateral contractility, apical expansion, and epithelial buckling leading to invasion into the adjacent tissue.^85^ Previous work suggested that myosin phosphorylation at the apical cortex is important for follicle cell shape which is only partly dependent on Rok.^87^ Future work is required to determine whether GEK/MRCK and Rok function cooperatively, for example, in these cells.

In summary, an increasing body of evidence suggests that MRCK plays a major role in apical domain organization and morphogenesis, in different developmental and homeostatic contexts. The spatiotemporal functional relationship between MRCK and ROCK may be a major defining factor in the fate of the apical domain. Future work to understand the interplay between MRCK and ROCK signaling at the cell cortex, is therefore likely to provide insight into control mechanisms of cell and tissue morphogenesis. For example, it would be of interest to determine whether an antagonistic relationship affects tension gradients at the cortex to control apical domain development during epithelial polarity establishment and morphogenesis. Another important question is whether MRCK plays a role in controlling the apical cortex during tissue-specific functions by the mature epithelium. Since, MRCK and ROCK also possess the ability to function in both polarity and tumorigenesis, understanding their complex relationship is likely to provide important insight into cancer progression.
